# The Effect of Diabetes on the Perioperative Outcomes of Colorectal Cancer Surgery Patients

**DOI:** 10.1371/journal.pone.0167271

**Published:** 2016-12-01

**Authors:** Raymond Yap, Simon Wilkins, Margaret Staples, Karen Oliva, Paul J. McMurrick

**Affiliations:** 1 Cabrini Monash University Department of Surgery, Cabrini Hospital, Malvern, Victoria, Australia; 2 Department of Epidemiology and Preventive Medicine, School of Public Health and Preventive Medicine, Monash University, Victoria, Australia; 3 Monash Department of Clinical Epidemiology, Cabrini Hospital, Malvern, Victoria, Australia; University Hospital Oldenburg, GERMANY

## Abstract

There are approximately 1.3 million patients in Australia with diabetes. Conflicting reports exist in the literature as to the effect of diabetes on the outcomes of colorectal cancer patients. We hypothesized that patients with diabetes would have poorer perioperative outcomes, and that diabetes was an independent risk factor for both 30-day mortality and perioperative morbidity. The aim of this study was to assess the impact of diabetes on perioperative colorectal cancer surgery outcomes, as compared to a diabetes-free reference population, and to examine factors affecting perioperative risk. We conducted an analysis of a prospectively collected, clinician-led colorectal cancer database of patients from 2010–2015. Patients with diabetes were compared to patients without diabetes on a range of perioperative outcomes. Pearson χ-squared tests, Wilcoxon rank sum tests and t-tests were employed for univariate analyses. Confounding factors were controlled for by separate logistic and linear regression analyses. The Huber-White Sandwich Estimator was used to calculate robust standard errors. A total of 1725 patients were analysed over 1745 treatment episodes in the study period with 267 patients (268 episodes) with diabetes studied. Diabetes contributed to medical, surgical complications, and increased length of inpatient stay in univariate analyses. Multivariable analysis adjusted for variables independently associated with each outcome revealed that diabetes was an independent contributor to an increased risk of surgical complications, with no significant effect on medical complications, return to the operating room, 30-day mortality, or readmission within 30 days. In this study, where overall baseline morbidity and mortality levels are low, the effect of diabetes alone on perioperative surgical outcomes appears to be overstated with control of associated perioperative risk factors such as cardiac, renal and respiratory factors being more important.

## Introduction

It is estimated that 1.3 million patients in Australia and a further 33 million patients in the United States have diabetes [1]. The proportion of these thought to be undiagnosed could be in the range of 40% [1]. Up to 20% of surgical patients will have diabetes listed as a co-morbidity [2]. A recent systematic review examining survival and colorectal cancer had ranges of 3–57% of colorectal cancer patients being afflicted with diabetes [3]. This review concluded that patients with diabetes had higher all-cause and cancer-specific mortality, as well as poorer disease-free survival [3]. Research, however, is lacking in examining the effect of diabetes on perioperative morbidity and mortality in non-cardiac surgery patients. It is postulated that patients with diabetes are at higher risk of perioperative mortality and morbidity [4]. This higher risk is often attributed to their microvascular and macrovascular pathology. Diabetes can induce a pro-thrombotic state [2], which may influence perioperative outcomes. There is also speculation that hyperglycemia in itself may be an underlying factor leading to poorer outcomes [5]. The function of leukocytes has shown to be adversely affected under hyperglycemic conditions [6, 7], and numerous studies have shown that diabetes is a factor for increased risk in surgical wound infections [8–10]. This effect on poor wound healing is probably through inhibition of various mechanisms, such as inhibition of keratinocyte migration, reduced fibroblast proliferation and over-expression of c-myc [11–13].

Three previous studies investigated morbidity and mortality in patients with diabetes undergoing non-cardiac surgery procedures, two of which used retrospective administrative databases, and one used a national cancer registry. Yeh et al., [14] used a nationwide database from Taiwan comparing diabetic to non-diabetic patients and found that patients with diabetes were at higher risk of perioperative mortality, as well as at higher risk of acute renal failure and acute myocardial infarction. Fransgaard et al., [15] found an increased 30-day mortality for patients with diabetes but no increase in post-operative complications. Anand et al., [16] using the United States Nationwide Inpatient sample database, concluded that patients with diabetes had a 23% lower mortality and fewer perioperative complications compared to patients without diabetes.

These conflicting results are difficult to reconcile. It is unclear why these three studies vary so much in their results and conclusions. Anand et al., [16] and Fransgaard et al., [15] only examined patients undergoing colorectal resection, while Yeh et al., (13) examined all non-cardiac surgery patients. It may be that Anand et al., (14) had a significant number of patients that were classified as diabetic patients, but in fact had sub-clinical diabetes. We note that Yeh et al., (13) attempted to control for this by including only patients who had at least one hospital admission and one outpatient visit for diabetes in the previous 24 months. However, this may have selected out a cohort at higher risk who would have other significant co-morbidities. Fransgaard et al., [15] based their diagnosis of diabetes on a combination of a national patient register and a national prescription registry. No differences were found between the different diabetic medication groups (metformin, oral diabetic medication, insulin and diet control) in mortality or morbidity. Nonetheless, these three studies provide a confusing mismatch of results into the effect of diabetes on perioperative outcomes.

This study aims to investigate the perioperative morbidity and mortality outcomes of diabetic and non-diabetic colorectal cancer patients using a high quality, prospectively maintained, clinical colorectal cancer database [17]. It is hypothesized that patients with diabetes would have poorer perioperative outcomes, and that diabetes was an independent risk factor for both 30-day mortality and perioperative morbidity.

## Materials and Methods

### Data Source

The Cabrini Monash University Department of Surgery colorectal neoplasia database is a high quality, prospectively maintained research database. This database incorporates all colorectal neoplasia data from the Cabrini Hospital and The Alfred Hospital, Melbourne and all operations are performed by specialist colorectal surgeons. This database has demonstrated very high levels of data completeness, accuracy, and patient follow-up due to clinician led data entry [17]. It includes detailed preoperative medical profiles, and combines this with detailed perioperative surgical information as well as pathological information. Patients are followed-up for a period of 5 years post-surgery, and all data on medical or surgical complications, including recurrence, are captured. The database also contains a comprehensive set of patient data including demographics, detailed pre-morbid conditions, type and manner of surgery, cancer staging, complications and follow-up. Ethics approval for this study was granted by the Cabrini Human Research Ethics Committee (Reference #11-22-06-15). Previous ethics approvals for this database are outlined in a published paper on the establishment on the database [17].

### Study Population

An analysis of the prospectively maintained Cabrini Monash University Department of Surgery colorectal neoplasia database was conducted. The selection criterion included all patients on the database between January 2010 and April 2015 who had undergone resection of colorectal neoplasia. The length of follow-up ranged from just over five years for patients having surgery in January 2010 to three months for patients from April 2015.

### Measures and Definitions

Due to the clinician-led nature of the database, all entries have been verified by clinicians undertaking primary care of the patients and are verified at a fortnightly data cleansing meeting that confirms veracity of outcome measures. Basic demographic data such as age and gender were included. Co-morbid conditions that are recorded were obesity (indicated by Body Mass Index (BMI)), hypertension, ischemic heart disease, angina, chronic cardiac failure, use of an anti-platelet agent, liver disease, chronic respiratory disease, cardiovascular disease, current or ex-smoker, and chronic renal impairment or dialysis. The primary outcomes were in-hospital and outpatient 30-day mortality, and surgical or medical complications. The division of complications into surgical and medical categories was previously developed by expert opinion and was adopted by the Colorectal Surgical Society of Australia and New Zealand for their Bi-National Colorectal Cancer Audit [18]. Secondary outcomes included rates of return to the operating room, 30-day readmission, and length of stay.

Diabetic status was determined by the separate field allocated in the database. The data includes the diabetes type, use of oral hypoglycemics or insulin, time since diagnosis of diabetes, insulin dose, and the presence of diabetes-related complications (ischaemic heart disease, peripheral vascular disease, neuropathy). Further comparisons were made between diabetic patients with and without diabetic-related complications.

Individual patient characteristics were summarised according to diabetic status. Data on patient demographics, perioperative risks, treatment, mortality and morbidity were compiled. Primary and secondary outcomes were examined for both groups.

### Statistical Analysis

For patients having more than one procedure during the period of the study, the characteristic at the time of the first procedure was reported. Differences between the groups were assessed with Pearson’s χ-squared tests and t-tests. Factors associated with the dichotomous outcomes of surgical complications, medical complications, return to the operating room, readmission to hospital within 30 days and 30-day mortality were investigated using separate logistic regressions for each outcome. Factors associated with length of stay were investigated using linear regression. The multivariable models for the association of diabetes with each outcome were adjusted for variables showing an independent association with the outcome. An independent association was defined a priori as a term that was significant at the 5% level in the multivariable model. A different set of adjustment variables was used for each outcome. Because some patients had more than one procedure during the period of the study, to account for the lack of independence between these observations, robust standard errors were calculated using the Huber-White Sandwich Estimator as implemented in the statistics software package Stata [19]. Data were analysed with Stata 13 (StataCorp LP, College Station, TX, USA).

## Results

A total of 1725 patients were analysed over 1745 treatment episodes in the study period. Twenty patients had metachronous cancers within the study period. Of the total study cohort, 267 patients had diabetes (268 surgical episodes), while 49 of these patients had one or more diabetes- related complication. Table 1 outlines the demographic and pre-morbid features of the patients, divided into patients with and without diabetes. Patients with diabetes were significantly older than patients without diabetes (median 75.7 vs. 69.6 years) and a higher proportion were male (Table 1). A higher proportion of patients with diabetes had colon cancer and had smoked previously. Patients with diabetes were in poorer health, with statistically significant higher rates of cardiovascular disease, stroke, respiratory disease, hypertension, chronic renal failure, and peripheral vascular disease, as well as a higher mean body mass index than patients without diabetes (Table 1).

**Table 1:** Demographics and pre-morbid conditions.

Feature	No diabetes	%	Diabetes	%	P-value
Patients	1458		267		
Treatment episodes	1477		268		
Gender					<0.001
- Male	697	47.8	174	65.2	
- Female	761	52.2	93	34.8	
Median age	69.6		75.7		<0.001
- (range)	(18.4–100.4)		(34.1–92.5)		
Colon cancer	999	68.5	202	75.7	0.02
Rectal cancer	459	31.5	65	24.3	0.02
Neoadjuvant treatment	219	15.0	29	10.9	NS
Median BMI^a^	25.3		27.7		<0.001
- (range)	(14.1–66.6)		(18.9–55.2)		
- < 20	119	8.2	4	1.5	
- 20–24.9	515	35.3	65	24.3	
- 25–29.9	467	32.0	74	27.7	
- 30–39.9	215	14.7	76	28.5	
- >= 40	16	1.1	8	3.0	
- not available	145	9.9	41	15.4	
PVD^b^	55	3.8	37	13.9	<0.001
Stroke	93	6.4	26	9.7	0.046
Ex-smoker	662	45.4	148	55.4	0.003
Current smoker	101	6.9	17	6.4	NS
Hypertension	606	41.6	192	71.9	<0.001
Chronic renal failure	39	2.7	31	11.6	<0.001
Respiratory disease	190	13.0	53	19.9	0.03
AMI^c^	69	4.7	26	9.7	0.001
Angina	28	1.9	13	4.9	0.004
Congestive cardiac failure	31	2.1	23	8.6	<0.001
Arrhythmia	136	9.3	51	19.1	<0.001

^a^BMI = Body Mass Index,

^b^PVD = Peripheral vascular disease,

^c^AMI = Acute myocardial infarction;

NS = Not statistically significant

Table 2 stratifies study patients with diabetes according to the type of diabetes, duration of diabetes, presence of complications and treatment. The vast majority of patients (262, 98.1%) had type 2 diabetes, with 46 (17.2%) requiring insulin as part of their treatment. Forty-nine (18.4%) of the patients had at least one diabetes-related complication and 69.7% were receiving oral hypoglycemics.

**Table 2:** Characteristics of diabetic patients.

Feature	N	%
Total	267	
Type 1 diabetes	5	1.9
Type 2 diabetes	216	80.2
Type 2 diabetes—ID^a^	46	17.2
Mean duration, years (range)	10.9 (0–47)	
Mean insulin dose, IU^b^ (range)	50.9 (10–150)	
Oral hypoglycaemic use	186	69.7
Complicated diabetes	49	18.4

^a^ID = Insulin dependent,

^b^IU = international unit

Table 3 describes the surgical features of the study patients. The with and without diabetes patient groups had some similarities such as rates of emergent and elective surgeries, as well similar proportions of the different surgical procedures for colon and rectal cancers. Patients with diabetes had more open procedures and a higher rate of laparoscopic to open conversions (11.2% vs. 6.9%, p = 0.02, overall Pearson’s χ-squared test). Patients with diabetes scored significantly higher on the American Society of Anaestheologists (ASA) score (ASA 3 and 4; 64.1% vs. 32.4%, p<0.001, Pearson’s χ-squared test).

**Table 3:** Surgical features of study patients.

Feature	No diabetes	%	Diabetes	%	P-value
Surgical urgency					NS
- Emergency	58	4.0	5	1.9	
- Urgent	86	5.9	20	7.5	
- Elective	1333	91.4	243	91.0	
ASA^a^					<0.001
- 1	343	23.5	9	3.4	
- 2	659	45.2	88	33.0	
- 3	419	28.7	150	56.2	
- 4	54	3.7	21	7.9	
- 5	2	0.1	0	0.0	
Surgical entry					0.02
- Open	460	31.6	93	34.8	
- Laparoscopic	787	54.0	126	47.2	
- Hybrid	94	6.4	18	6.7	
- Laparoscopic -> open	100	6.9	30	11.2	
- Robotic	35	2.4	1	0.4	
- TA-TME^b^	1	0.1	0	0.0	
Procedure type					NS
- Right hemicolectomy	502	34.4	105	39.3	
- Left hemicolectomy	72	4.9	16	6.0	
- Total colectomy	33	2.3	5	1.9	
- Subtotal colectomy	58	4.0	11	4.1	
- Proctocolectomy	18	1.2	2	0.7	
- High anterior resection	244	16.7	53	19.9	
- Low anterior resection	146	10.0	22	8.2	
- Ultra low anterior resection	269	18.4	32	12.0	
- Abdominoperineal resection	63	4.3	8	3.0	
- Hartmann's procedure	48	3.3	11	4.1	
- Other	25	1.7	3	1.1	

^a^ASA—American Society of Anaesthesiologists score,

^b^TA-TME—Trans-anal total mesorectal excision;

NS = Not statistically significant

The pathological features of both study patient groups are described in Table 4. There were no differences between the groups in terms of pathological diagnosis, lymphovascular invasion, and whether patients underwent radiotherapy. Patients without diabetes demonstrated a much higher rate of poorly differentiated cancers compared with patients with diabetes (20.7% vs. 5.9%, p = 0.03, Pearson’s χ-squared test), and had a significantly higher rate (34.7% vs. 27.7%, p = 0.03) of patients undergoing chemotherapy. Although there was a difference in overall cancer stage between the groups, there was no underlying trend towards a higher or lower stage.

**Table 4:** Pathological features of study patients.

Feature	No diabetes	%	Diabetes	%	P-value
Histological diagnosis					NS
- Adenocarcinoma	1127	77.3	214	80.1	
- Mucinous adenocarcinoma	142	9.7	30	11.2	
- Signet cell carcinoma	13	0.9	4	1.5	
- No residual	106	7.3	8	3.0	
- Dysplastic adenoma	84	5.8	12	4.5	
- Other	5	0.3	0	0.0	
Differentiation					0.03
- Well differentiated	51	3.7	38	14.8	
- Moderately differentiated	890	63.9	192	75.0	
- Poorly differentiated	288	20.7	15	5.9	
- Undifferentiated	11	0.8	1	0.4	
- Not assessed	153	11.0	10	3.9	
Overall stage					0.003
- Stage 0	229	15.7	22	8.2	
- Stage 1	229	20.5	56	21	
- Stage 2	396	27.2	97	36.3	
- Stage 3	368	25.3	68	25.5	
- Stage 4	165	11.3	24	9	
Lymphovascular invasion	453	32.5	78	30.5	NS
Adjuvant chemotherapy	484	34.7	71	27.7	0.03
Adjuvant radiotherapy	5	0.4	0	0.0	NS

NS, Not statistically significant

Table 5 compares the outcomes of patients with and without diabetes by univariate and multivariable logistic regression analyses. The presence of diabetes was associated with a significantly increased risk of surgical complications (Odds Ratio (OR) 1.45 CI 95% 1.05–1.99), and medical complications (OR 1.67, CI 95% 1.13–2.46), and increased length of stay (1.53 days, CI 95% 0.31–2.75) on univariate analysis. Multivariable analysis adjusted for variables independently associated with each outcome revealed that diabetes was an independent contributor to an increased risk of surgical complications, with no significant effect on medical complications, return to the operating room, 30-day mortality, or readmission within 30 days. The adjustment variables for each outcome are indicated below Table 5.

**Table 5:** Outcomes of study patients.

Feature	No diabetes	%	Diabetes	%	Unadjusted OR (95% CI)	Adjusted^a^ OR (95% CI)
Surgical complications	237	16.1	58	21.6	1.45 (1.05, 1.99)	1.44 (1.02, 2.04) ^a^
Medical complications	133	9.0	38	14.2	1.67 (1.13, 2.46)	1.10 (0.73, 1.68)^b^
Return to the operating room	69	4.7	19	7.1	1.56 (0.92, 2.63	1.08 (0.57, 2.05)^c^
30-day mortality	7	0.5	4	1.5	3.18 (0.93, 10.95)	2.47 (0.61, 10.1)^d^
Readmission within 30 days	130	8.9	30	11.4	1.26 (0.77, 2.06)	1.33 (0.85, 2.08)^e^
Inpatient death	9	0.6	6	2.2	1.13 (1.32, 10.58)	2.46 (0.83, 7.26)^f^

^a^—adjusted for sex, BMI, rectal cancer and operative urgency

^b^—adjusted for age, ischemic heart disease and ASA score

^c^—adjusted for age, surgical complications and medical complications

^d^—adjusted for ASA score and medical complications

^e^—adjusted for rectal cancer, surgical complications and medical complications

^f^—adjusted for surgical complications and medical complications

CI, confidence interval; OR, Odds Ratio.

A further analysis based on diabetic patients with and without diabetic-related complications was performed (Table 6). The presence of diabetic-related complications was significantly associated with an increased 30-day mortality (OR 13.7, 95% CI 3.4–54.7), and an increased length of stay (linear regression coefficient 3.8 days, 95% CI 0.7–7.1). There was a trend to increased surgical and medical complications, as well as an increased rate of return to the operating room, although these results were not significant.

**Table 6:** Outcomes of patients with diabetes with and without complications.

Feature	OR (95% CI)	P-value
Surgical complications	1.9 (0.9–3.6)	0.2
Medical complications	1.7 (1.0–3.8)	0.06
Return to the operating room	1.3 (0.4–4.4)	0.6
30-day mortality	13.7 (3.4–54.7)	<0.001
Readmission in 30 days	0.7 (0.2–2.8)	0.6
Length of Stay, mean (days)	^a^	0.02

^a^Linear regression coefficient 3.8 (0.5–7.1). CI, confidence interval; OR, Odds Ratio.

The predictive factors influencing increased surgical complications in the study patients are shown in Table 7. Body mass index (OR 1.04, 95% CI 1.01–1.06), emergency surgery (OR 3.04, 95% CI 1.54–5.98), rectal cancer (OR 2.14, 95% CI 1.36–3.36), and four surgical procedures (total colectomy, sub-total colectomy, abdominoperineal resection (APR) and Hartmann’s procedure) were predictive factors in the patient cohort for surgical complications. The surgical and medical complications found in patients in this series are listed in Table 8.

**Table 7:** Predictive factors of surgical complications.

Predictor variable	OR	95% CI	P-value
Diabetes	1.49	1.05–2.12	0.026
Body Mass Index	1.04	1.01–1.06	0.004
Urgency			
- Elective (Reference Group)	1		
- Urgent	NS	NS	NS
- Emergency	3.04	1.54–5.98	0.001
Rectal cancer	2.14	1.36–3.36	0.001
Surgical Procedure			
- Right or extended hemicolectomy (Reference Group)	1		
- Total and sub-total colectomy	2.32	1.34–4.02	0.003
- APR^a^ and Hartmann’s	1.87	1.05–3.32	0.035

^a^APR; abdominoperineal resection. CI, confidence interval; NS, Not statistically significant; OR, Odds Ratio.

**Table 8:** List of Surgical and Medical Complications.

Surgical Complications	Medical Complications
Abdominal/pelvic collection	DVT/PE^a^
Anastamotic leak	Chest infection
Enterocutaneous fistula	Cardiac
Superficial wound dehiscence	Other
Deep wound dehiscence	
Wound infection	
Sepsis	
Prolonged ileus	
Small bowel obstruction	
Urinary retention	
Ureteric injury	
Postoperative haemorrhage	
Other	

^a^DVT/PE, deep vein thrombosis/pulmonary embolism.

## Discussion

Our results indicate that although the outcome of patients with diabetes was worse than patients without diabetes, this was not significant after adjusting for other factors except in the area of surgical complications. Patients with diabetes had significantly higher co-morbidities than patients without diabetes. Therefore, it is likely that the myriad of co-morbidities that patients with diabetes have contributed more appreciably to their perioperative risk rather than the diabetes itself. This may help explain the conflicting findings of previous studies by Yeh et al., Anand et al., and Fransgaard et al., [14–16]. Although these previous studies have used logistic regression to try and control for confounding variables, there may be an unseen variable that is not apparent in the administrative databases used. Certainly, none of these studies control for impact and type of surgery. The finding in this study in relation to patients with diabetes and surgical complications is likely to relate to the higher rates of infection and poorer healing seen in patients with diabetes [8].

This study contrasts with the study by Anand et al., [16] that found that patients with diabetes have a 23% lower risk of mortality and lower morbidity following colorectal resection. In addition this study does not corroborate with Yeh et al., [14], which found that diabetes independently increases the perioperative risks to patients. To further conflict with these results, Fransgaard et al., [15] found no increased perioperative morbidity, but an increase of 17% in 30-day mortality in patients with diabetes. By including more variables into our multivariate analysis, we have been thorough in removing possible confounding factors in addition to ensuring that all surgeries were conducted with a specialist colorectal surgeon.

This study and previous studies, have adjusted for age, gender, and premorbid conditions. Additionally, our study adjusted for stage of disease and type of surgical procedure, absent from previous studies. Furthermore, the use of a high quality clinical database in this study minimises the possibility of a systematic error that may be present when using coding databases [14, 16].

Patients with ‘minor or borderline diabetes’ had been excluded in the study by Yeh et al., [14]. This has not been strictly defined on biochemical or clinical grounds, but rather on whether patients have had inpatient admissions or outpatient visits [14, 20, 21]. This non-standard definition also increases the risk that the results attributed to diabetes may be caused by an unseen factor, due to the diabetic patients who required recent admission often being frailer overall. Yeh at al., [16] attempted to mitigate for this using a matched system, although this does reduce the protective factor of randomization.

The quality of and access to preoperative and postoperative physician input to care may have been a factor in determining postoperative outcomes. This factor has been mitigated in our study as all patients received preoperative medical assessment and workup at either a private and or a public hospital. In addition, patients had postoperative medical assessment during their stay in hospital.

There are however some limitations to this study. Firstly, the numbers involved do not match some of the administrative-coding based studies previously published and therefore may underestimate any clinical effect of diabetes. However, the use of a high quality, clinically orientated prospective database in this study is invaluable and would be more accurate due to the involvement of clinicians in the data compilation. Secondly, the diagnosis of diabetic complications and severity are based on clinical data, rather than laboratory data such as HbA1c or fasting glucose levels. Thirdly, the data are derived from two tertiary level hospitals, and the results should be viewed from that perspective. Fourthly, the number of patients with Type 1 diabetes in this series is very low, even though a significant number of patients were classified as insulin-requiring Type 2 diabetes patients.

In this study diabetes is a marker for an increased risk of postoperative morbidity and increased length of stay, however it only remained an independent contributor to the risk of surgical complications. One of the difficulties inherent to the study of diabetic patient outcomes is that many of the premorbid conditions such as chronic renal failure and ischaemic heart disease are directly influenced by diabetes, and indeed are a marker of severity and duration of diabetes. These same conditions have adverse effects on perioperative outcomes, which can be independent of the presence of diabetes. This was reflected in our study with ischaemic heart disease being an independent predictor of medical complications. Although patients with complications of diabetes did more poorly on one measure (increased 30-day mortality), the other areas such as surgical and medical complications were not significant. This is most likely due to the fact that different complications such as ischaemic heart disease and neuropathy have different effects on the surgical outcome, as well as the smaller numbers in this cohort. Therefore, this study points out that the long-term control of diabetes to reduce the incidence and severity of these associated pre-morbid conditions is probably more important than the short-term control of perioperative hyper- and hypo- glycaemia. Although outcomes such as wound infection have had direct association with hyper- and hypo- glycaemia [8–10], this area is not well studied. This adds another argument to effective long-term control of diabetes; that is, the improvement of perioperative outcomes. Clinicians and patients should realise that a short-term improvement in diabetic control peri-operatively is unlikely to reduce perioperative risk. Further research targeting the perioperative effects of hypoglycaemia and hyperglycaemia is required.
